# Distinct actin architectures across a giant syncytial cell

**DOI:** 10.17912/micropub.biology.002421

**Published:** 2026-09-20

**Authors:** Andrew D. Countryman, Zachary M. Geisterfer, R. Christine Cao, Amy S. Gladfelter

**Affiliations:** 1 Department of Biomedical Engineering, Columbia University, New York, NY, United States; 2 Physiology: Modern Cell Biology Using Microscopic, Biochemical and Computational Approaches, Marine Biological Laboratory, Woods Hole, MA, United States; 3 Department of Cell Biology, Duke University, Durham, NC, United States

## Abstract

The syncytial slime mold *Physarum polycephalum* exhibits cytosolic streaming, cell migration, and cell network remodeling within a single cell. It is unclear how cytoskeletal elements are spatiotemporally organized to mediate these processes. Here, we combine live and fixed imaging of *Physarum* F-actin to uncover organizational and dynamic signatures of the actin cytoskeleton across cell structures. We find circumferential actin filaments constrict and relax to drive peristaltic contractions in feeding tubes, while migratory fans are characterized by actin patches and comets. Our work presents undescribed actin structures in *Physarum* and demonstrates that different actin-based behaviors coexist within a contiguous cytoplasm.

**Figure 1. Actin ultrastructure varies across a single Physarum polycephalum macroplasmodium:** **(A)** Brightfield image of the macroplasmodium of Physarum polycephalum, with the feeding tubes (blue box) and the migratory fan (red box) marked. Scale bar = 1 mm. **(B)** Brightfield images with inverted LUT showing the expansion of a feeding tube during peristaltic contractions. Scale bar = 50 µm. **(C)** Fixed, phalloidin-labeled actin structures in the feeding tube. Color coding indicates z-depth (see color scale bar). Scale bar = 50 µm. **(D)** Fixed, phalloidin-labeled actin structures in a migratory fan. Color coding indicates z-depth (see color scale bar). **(E)** Actin assemblies visualized through LifeAct-GFP in a live feeding tube. 3D images taken from Extended Data Movie 1. Color coding indicates z-depth (see color scale bar). Scale bar = 50 µm. **(F)** Actin structures, labeled with LifeAct-GFP, in a live feeding fan. 3D images taken from Extended Data Movie 1. Color coding indicates z-depth (see color scale bar). Scale bar = 50 µm. **(G-I)** Image of a feeding tube costained with phalloidin (magenta) and Hoechst (blue). Scale bar = 15 µm. Inset scale bar = 5 µm.

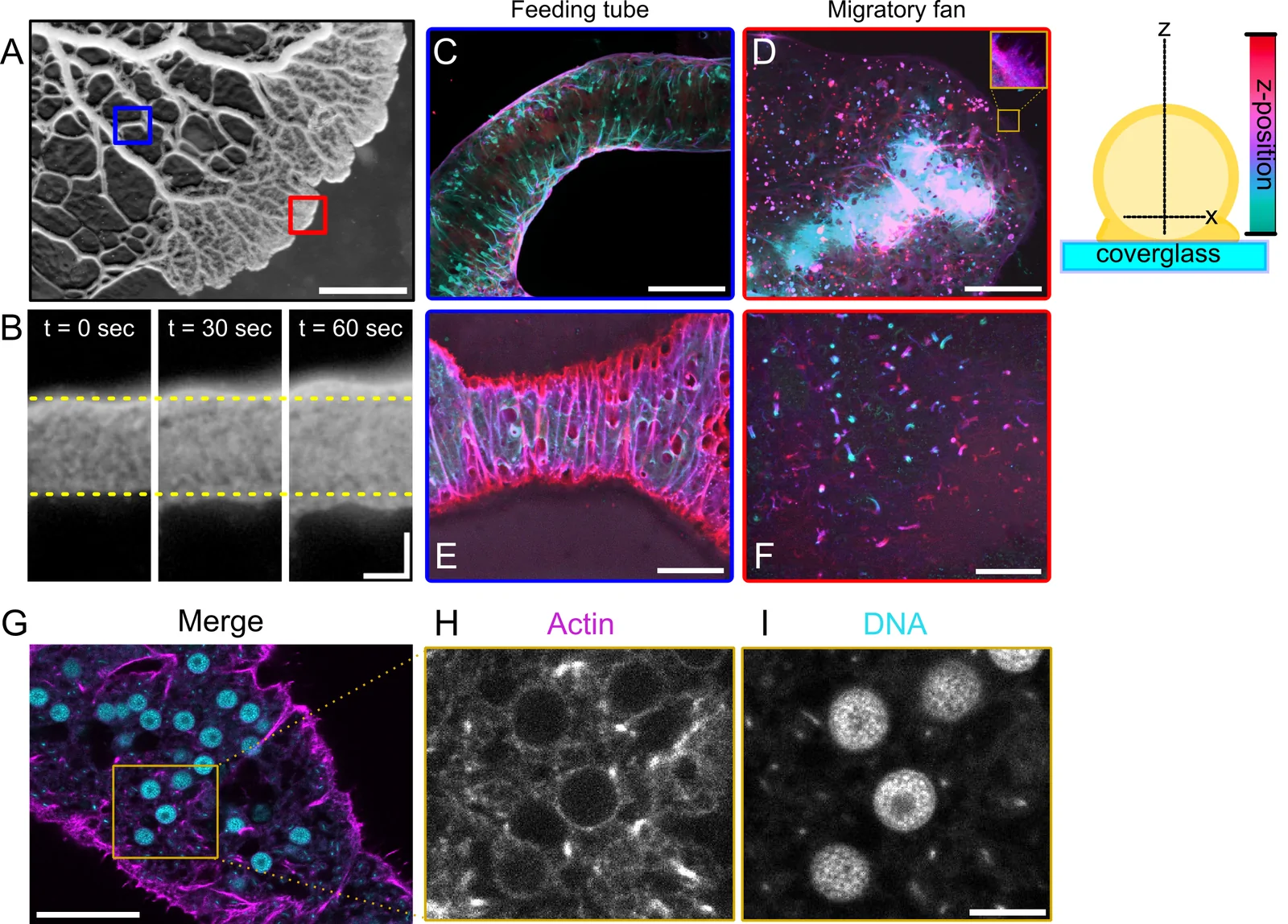



## Description

The macroplasmodial stage of the acellular slime mold *Physarum polycephalum* is a syncytial, reticulated network, with millions of nuclei inhabiting a shared cytoplasm (Fig. 1; Dove et al., 1986). The cell uses long-range cytoplasmic streaming, with velocities on the order of millimeters per second, to transport bulk material across centimeter-scale intracellular distances ( Fig. 1A, B; Kamiya 1961). These substantial cytoplasmic flows are driven by actomyosin-based peristaltic contractions of the cell network, although the molecular and regulatory nature of these contractions remains poorly understood ( Fig. 1B ; von Olenhusen & Wohlfarth-Bottermann 1979, Kamiya 1981, Alim et al., 2013). Flows transport nutrients and molecular signals across different parts of the polarized cell, from large tube structures at food sources to fan structures at migratory fronts ( Fig. 1A ; Kramar & Alim 2021). It remains unclear how *Physarum* compartmentalizes and/or coordinates different actin-based mechanical and structural behaviors across tube and fan structures within a single cytoplasm.

To visualize actin structures across *Physarum* macroplasmodia, we used two parallel approaches: fixed imaging of F-actin via phalloidin staining and live imaging of F-actin via injection of LifeAct-GFP. We grew macroplasmodia on fresh water-agar, then selected and excised large regions of the cell containing both fan and tube structures. These were then either microinjected with LifeAct- GFP or fixed and stained with phalloidin. Below, we characterize in more detail the distinct actin structures found in the tube and fan regions of fixed and live *Physarum* macroplasmodia.

First, we describe actin structures in feeding tubes. In fixed, phalloidin-labeled tubes, actin was primarily assembled into a sheet-like cell cortex and into strand-like circumferential filaments perpendicular to the long axis of the feeding tube, as previously characterized ( Fig. 1C, teal; Oettmeier et al., 2018). Live imaging of tubes injected with LifeAct-GFP confirmed the presence of both sheet-like cortical ( Fig. 1E, teal) and filamentous circumferential ( Fig. 1E, magenta) actin structures. In these live specimens, we found that during every contraction/relaxation cycle, the circumferential filaments stretched and shortened while retaining their shape and position (Extended Data Movie 1, left). This finding differs from previous electron microscopy-based characterization of contraction-linked actin structures in *Physarum* , which emphasized a mechanical function of longitudinal filaments, suggested that filaments fluctuated between linear and mesh-like structures to drive each contraction cycle, and posited a role of dynamic actin filament assembly and disassembly (Nagai et al., 1978, Fleischer & Wohlfarth-Bottermann, 1975). Our fixed and live imaging clarify that circumferential, rather than longitudinal, filaments are the dominant actin structure in tubes and argue against a role of actin network disassembly or restructuring in modulating tube contraction state. This suggests that contraction may instead be controlled by regulated myosin activity, which can generate and relax tension via sliding of adjacent actin filaments without requiring complete network remodeling (Muresan et al., 2022, Quintanilla et al., 2023).

In contrast to the filamentous actin structures in tubes, fixed, phalloidin-labeled fans were instead characterized by an actin cortex with additional patch-like structures ( Fig. 1D ). Additionally, we observed smaller protrusive structures in both fans (Fig 1D, inset) and tubes, reminiscent of filopodia and potentially marking sites of new cell branches. Live imaging of fans injected with LifeAct-GFP revealed diffuse cortical actin as well as dense, fast-traveling actin comets ( Fig. 1F ; Extended Data Movie 1, right), which were also seen to a lesser extent in tubes (Extended Data Movie 1, left). In one living sample, we noticed fan remodeling and retraction away from the area illuminated by microscope light (Extended Data Movie 1, right). During this remodeling event, we observed dense actin comets associated with a transition in actin structure from a sheet-like cortical morphology into filaments (Extended Data Movie 1, right, inset). In other contexts, actin comets are known to play roles in microbe locomotion and intracellular trafficking (Tilney & Portnoy 1989, Giardini et al. *,* 2003), but our observations here implicate them as potential mediators of actin remodeling. Together, both live and fixed imaging approaches suggest that actin structures in the fan region of the cell may be specialized to drive migration and endocytosis/exocytosis (Taunton et al., 2000) rather than cytosolic streaming, which has been attributed to the circumferential actin structures present in tubes.

To understand how nuclei were positioned with respect to the actin cytoskeleton in this syncytial cell, we co-stained fixed specimens with phalloidin and Hoechst. We found that nuclei were distributed throughout the cell, occupying phalloidin-depleted regions within an otherwise actin-rich cytoplasm ( Fig. 1G –I). While it is possible that actin may play a role in nuclear positioning, we did not observe any obvious, stereotyped actin structures associated with individual nuclei. Further studies will be necessary to explore the functional importance of actin in positioning organelles under substantial cytoplasmic flow.

Overall, our work unveils and clarifies the diverse mechanostructural roles of the actin cytoskeleton in the *Physarum polycephalum* macroplasmodium. The whole organism contains cortical actin, which hugs the invaginated cell membrane and likely serves to position organelles and provide structural support to the cell, as well as sparse peripheral filopodial protrusions, which have not yet been described but may serve to nucleate new cell branching events. While tubes are characterized by circumferential actin filaments that constrict to drive cytoplasmic streaming, fans instead display a patch-like organization and dense actin comets, which may be associated with dynamic network remodeling, migration, and secretion. We find that actin-based structures do not significantly change or remodel across a single contraction cycle, but can convert between cortical, filamentous, and comet-like morphologies on the scale of tens of minutes. Future work probing the functions of these newly described cytoskeletal elements and uncovering the regulatory pathways that organize such distinct structures within a single cell will prove informative.

## Methods

*Physarum polycephalum* macroplasmodia were grown on 1.7% (wt/vol) low-fluorescence water agar and fed oat flakes every two days. Twenty-four hours before imaging, 1 cm ^2^ agar blocks containing a region of the cell were transferred to a new agar plate and left to develop a mature network in the absence of oat flakes. After starving the sample for 24 hours to reduce autofluorescence, the samples were prepared for live or fixed imaging.

For fixed imaging, sections of the network were prepared by excising an agar block containing structures of interest and gently immersing the entire agar block and specimen into 4% formaldehyde for 10-15 min at RT. After fixation, samples were rinsed three separate times using RT PBS buffer. Each rinse was approximately the volume of the initial formaldehyde immersion and was left for 5 min to allow buffer exchange within the agar block. After rinsing the specimen, samples were immersed in PBS buffer containing either 660 nM Phalloidin-Rhodamine ( Fig. 1C, D) or 660 nM Phalloidin-Rhodamine and 500 nM Hoechst 33342 ( Fig. 1G –I) and incubated for 10 min at RT. The specimen was rinsed once in PBS as described above, and subsequently all buffer was removed from the imaging well containing the sample. 3D fluorescence microscopy was performed on a Nikon Ti2-E inverted microscope equipped with a Yokogawa CSU-W1 spinning disk confocal. Images were acquired using a 40x Plan Apo silicon immersion objective (NA = 1.25; Fig. 1C, D). Selected images were displayed as a z-depth colored intensity projection for Fig. 1C, D. For fixed imaging of actin and nuclei, fluorescence microscopy was performed on Zeiss LSM 980 confocal. Images were acquired using a 63x Plan-Apochromat oil immersion objective (NA = 1.4; Fig. 1G- I).

For live samples, agar blocks containing regions of interest were transferred to a stereomicroscope (Zeiss Stemi 305) equipped with a FemtoJet 4i microinjector (Eppendorf). Injection needles (World Precision instruments; OD=1.0mm, ID=0.75mm) were pre-loaded with ~3 μl of LifeAct-GFP (250 µM) and set to a shallow angle of 5-10° relative to the face of the agar block. The compensation pressure was set to 60 hPa, the injection time was set to 0.5 seconds, and the injection pressure was initially set to 80 hPa. The needle was delicately introduced to a larger tube section of the specimen where the LifeAct-GFP was released. Due to the large intracellular pressure variation within and between *Physarum* samples, the injection pressure was increased in intervals of 10 hPa until injection resulted in visible release of LifeAct-GFP into the cell. Then, the injection was sustained until reaching a final injection volume of approximately 200 nL. Samples were left to distribute the LifeAct-GFP briefly before imaging. Time-lapse fluorescence imaging was performed on a Nikon Yokogawa CSU-W1 spinning disk confocal using a 20x air objective ( Fig. 1E ; Extended Data Movie 1, left) or on an Olympus IXplore SpinSR spinning disk confocal using a 20x air objective ( Fig. 1F ; Extended Data Movie 1, right). A single time point from the imaging is displayed as a z-depth colored intensity projection for Figure 1 E,F.

## Reagents

Phytagel (Sigma-Aldrich P8169)

Old Fashioned Oats (Quaker)

Formaldehyde (Sigma-Aldrich P47608)

1x PBS (adjust to pH 7.8 with KOH)

Rhodamine Phalloidin (Invitrogen R415)

Hoechst 33342 (ThermoFisher H1399)

Glass capillaries (World Precision instruments TW100-3)

LifeAct-GFP (250 µM)
